# Editorial: Food and Nutrition Security: Underutilized Plant and Animal-Based Foods

**DOI:** 10.3389/fnut.2021.821388

**Published:** 2022-01-26

**Authors:** Olivia R. L. Wright, Dharini Sivakumar, Yasmina Sultanbawa, Michael E. Netzel

**Affiliations:** ^1^ARC Industrial Transformation Training Centre for Uniquely Australian Foods, Queensland Alliance for Agriculture and Food Innovation, The University of Queensland, Indooroopilly, QLD, Australia; ^2^School of Human Movement and Nutrition Sciences, The University of Queensland, St. Lucia, QLD, Australia; ^3^SARChI Research Chair in Phytochemical Food Network, Tshwane University of Technology, Pretoria, South Africa

**Keywords:** editorial, underutilized foods, biodiversity, sustainability, nutrition, health

There is an urgent need to address the issue of climate change. A transdisciplinary approach integrating the sciences and humanities is required to manage such a global challenge. The development of sustainable food systems is a vital component of climate change management. The current global food system is not viable in terms of health, affordability, or the environment—in other words, it is not sustainable. The triple burden of malnutrition is leaving billions of people undernourished worldwide. Paradoxically, there are also billions who are over-nourished, yet micronutrient deficient, due to the repeated consumption of energy dense, nutrient poor foods. There is a clear relationship between malnutrition and reliance on a few staple crops or low dietary diversity. Lack of dietary diversity has also been associated with inadequate intake and risks of deficiencies of essential micronutrients, including vitamin A, folate, iron, and zinc. Typically, this is seen in low-income households who subsist on staple-based diets, often produced through unsustainable food systems.

Phytochemicals in plant foods, otherwise known as non-nutritive compounds, are associated with health-promoting properties due to their essential function in biological mechanisms that maintain health and well-being. The growing understanding of the chemo-preventive properties of foods of plant origin has led to discussions on their potential contribution to daily diets or the inclusion of their constituents into dietary patterns for the prevention of non-communicable diseases.

A radical transformation of the food system as we know it is needed to enact positive changes globally and to impact food security. This Research Topic focuses on the investigation of alternate foods and sustainable food systems—in particular, underutilized plant and animal-based foods. The alternate foods and systems presented could lead to increased equity in food availability, affordability, and nutrition, especially for more vulnerable populations. Underutilized edible plants and animal-based foods used by Indigenous populations globally could play a key role in the development of such alternate food systems. These underutilized foods can be rich sources of macro- and micronutrients; some examples include legumes such as lentils and wattles which are high in protein, dietary fiber, and trace elements. They can be well-adapted to grow in arid and semi-arid conditions and are able to fix atmospheric nitrogen, which enriches soil fertility resulting in more sustainable agricultural and food systems. Similarly, there are many examples of underutilized roots and tubers, fruits and vegetables, and oil seeds that have been consumed by Indigenous communities for thousands of years. These have significant potential to contribute to dietary diversity and could be included in the diet of future generations. The sustainability of these systems is also strengthened by the inherent cultural practices of these communities with respect to land and water management, leading to sustainable food production.

Seventeen quality papers, 11 research articles, five reviews, and one perspective article, are published in this Special Issue. The topics that were researched and/or reviewed are: neglected and underutilized crop species: the key to improving dietary diversity and fighting hunger and malnutrition in Asia and the Pacific; the nutritional value of edible Crickets; consumer acceptance of insect-based protein; potential of farming Shipworms as a sustainable, nutritious, and affordable food source; seed composition and amino acid profiles for Quinoa grown in Washington State; diversity of essential oil profiles in Cardamom accessions from Southern India; bioactive components and radical scavenging activity in salt-tolerant Amaranth; metabolite fingerprinting of Kersting's Groundnut seeds; nutritional potential of the native Australian Green Plum; promising nutritional attributes of Samphire, an underutilized Australian indigenous edible halophyte; assessment of dietary bioactive phenolic compounds and agricultural sustainability of *Corchorus olitorius* L., an African leafy vegetable; interactions between phytochemicals and minerals in *Terminalia ferdinandiana* and implications for mineral bioavailability; potential of Bambara Groundnut for global food security and nutrition; the effect of irrigation, postharvest processing, and different household cooking techniques on the nutritional quality of African Nightshade and Chinese Cabbage; traditional food systems and indigenous food consumption in selected local Indian communities and existing challenges.

We hope that this Special Issue will further promote the interest in underutilized plant and animal-based foods and their crucial role in a diverse, sustainable, and healthy diet.

## Author Contributions

MN prepared the original draft which was reviewed and edited by OW, DS, and YS. All authors approved the submitted version.

## Conflict of Interest

The authors declare that the research was conducted in the absence of any commercial or financial relationships that could be construed as a potential conflict of interest.

## Publisher's Note

All claims expressed in this article are solely those of the authors and do not necessarily represent those of their affiliated organizations, or those of the publisher, the editors and the reviewers. Any product that may be evaluated in this article, or claim that may be made by its manufacturer, is not guaranteed or endorsed by the publisher.

